# Sustainable Management of the African Great Lake Coastal Areas: Motivations and Perspectives of Community Citizen Scientists

**DOI:** 10.1007/s00267-023-01824-x

**Published:** 2023-05-08

**Authors:** Happiness Anold Moshi, Daniel Abel Shilla, Joan Brehim, Ismael Kimirei, Catherine O’Reilly, Steven Loiselle

**Affiliations:** 1 Tanzania Fisheries Research Institute, Kigoma Centre P.O. Box 90, Kigoma, Tanzania; 2 Department of Aquatic Sciences and Fisheries Technology, University of Dar es Salaam, Dar es Salaam, Tanzania; 3 Department of Sociology and Anthropology, Campus Box 4660, Schroeder Hall 332, Illinois State University, Normal, IL 61790-4660 USA; 4 Tanzania Fisheries Research Institute, Dar es Salaam Headquarters, P.O. Box 9750, Dar es Salaam, Tanzania; 5 Department of Geography, Geology and the Environment, Illinois State University, Normal, IL USA; 6 Earthwatch Europe, 255 Banbury Road, Oxford, UK; 7 University of Siena, INSTM, Via Aldo Moro 2, Siena, Italy

**Keywords:** Citizen science, African Great Lakes, Lake Tanganyika, Motivation factors, Water quality, Sustainability

## Abstract

The long-term sustainability of the African Great Lakes is strongly connected to the management and monitoring of their coastal areas. Yet, the communities that live in these areas are rarely involved in monitoring and have limited influence on key management issues. Furthermore, regulatory activities and knowledge sharing in these transnational ecosystems are strongly limited by funding and infrastructure limitations. Citizen science has great potential to advance both scientific and public understanding of the state of the environment. However, there remains a limited understanding of participants’ motivations and expectations, especially in developing countries, where citizen science has great potential to complement regulatory monitoring. The present study explores the motivations of citizen scientists in villages along Lake Tanganyika’s northern coast and their potential to take a more active role in lake management. Motivations were examined through qualitative interviews, focus groups, and quantitative surveys with 110 citizen scientists and 110 non-citizen scientists from participating villages. Key motivational factors identified were the desire to contribute to scientific research and local knowledge, as well as aspects of financial compensation. The results confirm that participation in citizen science provides many benefits to participants beyond their role as data aggregators and final knowledge users. However, the incentives to participation varied to those typically considered in citizen science programs conducted in developed countries. To create sustainable long-term community based environmental monitoring, these motivations should be incorporated in the program design and participant recruitment.

## Theoretical Background

Citizen science, the participation of non-scientists in the monitoring and assessing the environment, has been growing rapidly since the environmental revolutions of 1960’s to 1970’s (Roy et al., 2012). According to Bela et al. (2016), the most important benefit of involving citizen science in environmental research is to create a strong tie between local and formal impacts of scientific research. In citizen science, community members may participate in different aspects of research including designing project goals, data collection, analysis and report writing (Bonney et al., 2009). This opens up new possibilities to collect and process large quantities of data across a diverse habitats and locations over long spans of time; on a scale that traditional research often cannot attain (Hadj-Hammou et al., 2017). Citizen science has been used successfully to monitor aquatic environments in many parts of the world (Hughes et al., 2014; Hyder et al., 2017; Loiselle et al., 2016; Moshi et al., 2022; Thornhill et al., 2019). Studies focused on assessing citizen science for sustainable development goals reporting (Fritz et al., 2019; Bishop et al., 2020, Fraisl et al. 2020), evaluating ecosystem services (Di Grazia et al., 2022), monitoring litter (Mayoma et al., 2019) or coliform pollution (Moshi et al., 2022) show that citizen science can meet a wide range of objectives. However, in Africa and many developing counties, the field of citizen science has so far been limited and focused on aspects such as hydrology (Njue et al., 2019; Rufino et al., 2018; Weeser et al., 2018).

Citizen science shows a great potential in advancing both scientific research, decision and policy support as well as public understanding of science (Schade et al. 2021). However developing successful citizen science projects require a clear understanding of citizen scientists’ perceptions and motivations (West et al., 2021). These perceptions and motivations can vary from location to location, and in relation to social and economic background, with very limited information on citizen scientist involvement from developing countries and marginalized communities (Asingizwe et al., 2020; Walker et al.^2021^, Wright et al. 2015).

In general, citizen scientists have different motivations for participating in environmental activities than people who participate in other social activities (Seeberger, 2014).. Without understanding motivation behind citizens’ participation in citizen science projects, citizen science programs can have multiple challenges to recruit and maintain participation in citizen science program that require continued or long-term participation (Alender, 2016).

An individual’s motivation for participating in citizen science has been described as egoistic, altruistic, or both (Kragh, 2016). Ryan et al. (2001) found that altruistic aspects like improving the environment were most important for citizen scientists three programs in North America. Altruistic motives were also found to be important in participants of online citizen science (Galaxy Zoo) (Raddick et al., 2013a). Contributing to science was also found to be a major motivation (Domroese and Johnson, 2017; West and Pateman, 2016). Other citizen scientists were driven by environmental values (Curtis, 2015; Measham and Barnett, 2008) while some citizen science participants were motivated more by social aspects (Asah et al., 2014).

Citizen scientists’ expectations from participation can also vary significantly (Ganzevoort et al., 2017), and can include more egoistic aspects such as financial compensations and individual learning and understanding (Beza et al., 2017; Paul et al., 2020). The provision of incentives to citizen scientists, such as prizes, certificates, workshops and meetings, can increase motivation (Lewandowski and Oberhauser, 2017; Luther et al., 2009; Restuccia et al., 2016).

Many citizen science projects focus on ecology, biodiversity and conservation of resources but only few have explored the motivations of people in developing countries, where marginal communities have limited economic and political agency (Walker et al., 2021). Importantly, citizen science in these countries can make a major contribution to improve environmental monitoring and management (Bishop et al. 2020). Understanding what motivates local communities and people to participate in a citizen science project can increase the possibility of accurate long-term data collection necessary to support resource management, as well as improve local attitudes towards a more sustainable resource use (Ganzevoort et al., 2017, Sauermann et al., 2020; Sutherland et al., 2015; West and Pateman, 2016).

The present study explores citizen motivations toward participation in a large-scale and long-term study of the coastal waters of Lake Tanganyika (Tanzania). This study is addressed at providing new insights to following research questions (i) which members of the community are most willing to participate in citizen science based water quality monitoring, (ii) what are their motivations and expectations, (iii) what incentives favor long-term participation.

## Methodology

### Description of the Study Area

Lake Tanganyika is the world’s longest freshwater lake located at 6.2556° S, 29.5108° E and the deepest (1470 m) of the African Great Lakes. The lake is shared between four countries, Burundi (8%), Democratic Republic of Congo (DRC) (45%), Tanzania (41%) and Zambia (6%) (Reynolds and Moelsae, 2000) and has a catchment of 231,000 km^2^. Despite of its depth and oligotrophic nature, the lake is threatened by pollution from domestic and industrial activities, climate change, land use changes and rapid increase in human population which has led to overexploitation of its aquatic resources and habitat destruction. Monitoring of the coastal areas of Lake Tanganyika, like many lakes in Africa is limited both in geographic scope as well as long-term continuity, usually in relation to limited and inconsistent funding (Nijhawan, Howard (2022)).

**Fig. 1:**
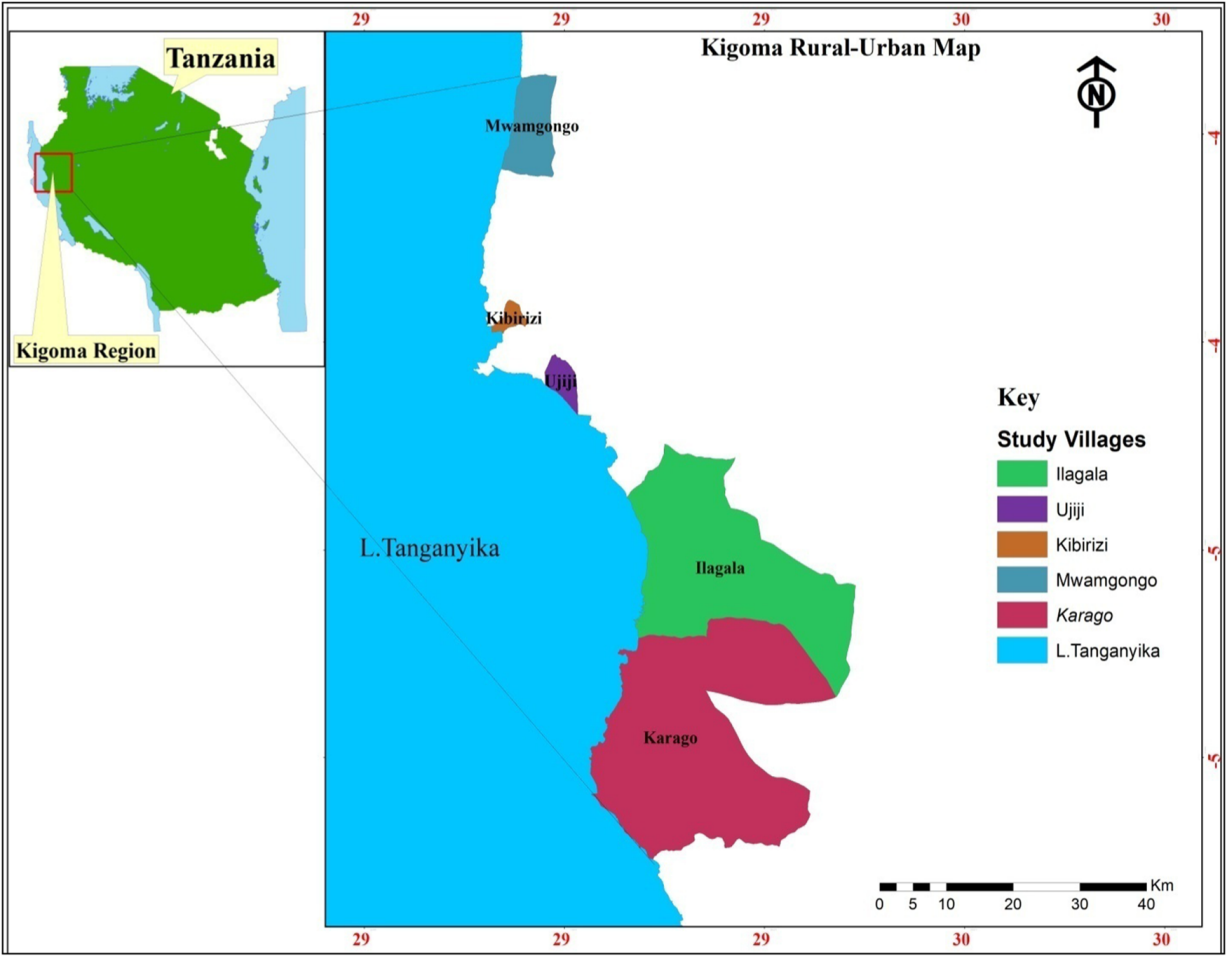
Map of Lake Tanganyika showing study sites

The present study was conducted in five lakeshore villages (Fig. 1), chosen based on differences in their population, rural/urban dominance and access to the lake for monitoring activities. The villages Karago and Mwamgongo (rural dominance), Ilagala, Kibirizi and Ujiji (urban dominance) have a range of livelihood activities, ranging from fisheries, agriculture, palm oil plantation, palm oil processing, fish processing to small businesses, with fisheries as the most important income generator and protein source. According to the 2012 national census, Ilagala village has a population of 18,087 people which is the larger compared to Mwamgongo (15,657), Kibirizi (12,225), Ujiji (9040) and Karago (5456) (NBS. (2013)).

### Citizen Science Water Quality Monitoring in Lake Tanganyika

A community-based citizen science water quality monitoring was initiated in 2018 by Tanzania Fisheries Research Institute (Kigoma Center) and initially funded by the EU 2020 MONOCLE project (Multiscale Observation Networks for Optical Monitoring of Coastal waters, Lakes and Estuaries).

The project aimed to collect water quality data in coastal areas to improve both national as well as local management of the coastal environment. 50 people, including fishermen, farmers, Beach Management Units (BMUs), fish processors, and fish traders, were randomly chosen from each village. Initial recruitment was facilitated by key informants in each village (e.g. fishery officers). Participation was limited to adults (over the age of 18) who were willing to perform water quality monitoring activities and who were available to do so. It should be noted that this selection criteria introduced some bias to participation, as persons who had no interest in participating or after completing a structured questionnaire, 150 out of 250 individuals (30 in each village) were recruited and trained to become citizen scientists.

The citizen scientists used the FreshWater Watch monitoring, training and quality control approach developed by Earthwatch Europe (Moshi et al., 2022; Thornhill et al., 2019). These trained citizen scientists began their engagement in the project in 2019 and have continued collecting, analyzing and recording water quality information once in a month to the present, with a recent expansion into 3 new villages. For each sampling month, citizen scientists are rewarded each an airtime voucher equivalent to 5000 Tanzania Shillings (2.15 US Dollars). The voucher was considered both an incentive as well as support for data transmission of the recorded measurements by smartphone. Water quality parameters monitored by citizen science include nutrient concentrations, bacterial load (coliforms), turbidity, watercolor, and total suspended solids, as well as hydrological and meteorological observations.

### Research Design and Data Collection Methods

Field work to understand the motivation factors for community participation was carried out from May to August 2020 in all five study sites. Data were collected through Focus Group Discussions (FGDs), Key Informant Interviews (KIIs) and individual questionnaire surveys. A mixture of these methods was used to cross check and validate the collected information. This study involved two groups of people; citizen scientists who are part of the water quality-monitoring project and non-citizen scientists who are not part of water quality monitoring project. The citizen scientist respondents had already participated in community water quality monitoring for more than one year. The group of non-citizen scientist respondents (who were not participating in water quality monitoring) were identified and selected via the snowball sampling approach, where the non-citizen scientist respondent (who is not participating in water quality monitoring) was identified by the citizen scientist respondent (Kothari, 2004; Marshall, 1996). This sampling techniques was selected due to the difficulty in reaching the number of potential respondents required, given the extension of the villages and the informal nature of participant daily activities (Woodley and Lockard, 2016). The effectiveness of this method has been used in a number of studies, typically for marginalized populations, allowing researchers to be introduced to participants that would be difficult to identify. It should be noted that there are a number of limitations to this approach, as contacts remain within a respondent’s social network, making the representativity of the control population very similar to that originally selected as citizen scientists (Cohen and Arieli, 2011).

Prior to the data collection, respondents were informed about the purpose of the study and their willingness to participate was requested.

#### Individual Questionnaire Survey

Prior to the actual survey, a pilot survey was conducted with a few individuals to test the relevance and clearness of the questionnaire. During the actual survey, the final semi-structured questionnaire, in Swahili, was administered to 110 citizen scientists and 110 non-citizen scientists. The sample size was based on the availability of citizen scientists and non-citizen scientists and their willingness to participate in the study and its suitability for statistical testing (Kotrlik and Higgins, 2001).

The questionnaire addressed information regarding participant socio-demographic characteristics and willingness to participate in future citizen science activities (Appendix 1). The questions regarding their motivations for participation were a combination of intrinsic and extrinsic motives adapted from Budhathoki and Haythornthwaite (2013) and modified on the basis of the information collected from the FGDs and KIIs. Statements regarding motivational factors (interaction with professional scientists, contribution to scientific research, providing/sharing information, hobby, interact with the community members, help the researcher do his/her research and expect something from the researcher) were assessed using a five-point Likert scale, from 1 = very low importance, 2 = low importance 3 = medium importance, 4 = high importance and 5 = very high importance (Appendix 1). Information regarding personal and social expectations for participating in citizen science activities, preferred incentives and ideas on approaches to ensure sustainability of citizen science activities were also collected during the survey (Appendix 1).

#### Focus group discussions and key informants’ interviews

Focus groups are often used to obtain consensus-based thoughts and opinions of people with similar backgrounds. Two groups of FGDs with 5–8 participants of mixed gender and age were held at the community level in each village to gather qualitative information related to the results of the questionnaire survey. Members of FGDs were selected from the individuals who participated in the questionnaire survey. In each village, one FGDs was organized with individuals who are not part of the water quality monitoring project (non-citizen scientists) and a second FGDs was composed of active citizen scientists engaged in water quality monitoring. A total of 10 FGDs were conducted.

Information regarding their environmental concerns, motivations for participating in monitoring activities, expectations, incentives and their ideas on how to ensure sustainability of the monitoring activities was discussed, following a pre-prepared checklist of questions (Appendix 2). The discussion was facilitated by the researcher. FGDs utilized open-ended questions which offered advantage of respondents to freely express their ideas.

Information provided by FGDs were cross-checked and validated by conducting in-depth KIIs. According to Trochim (2006), a key informant is an individual who is accessible, willing to talk and have detailed information and knowledge about a specific topic under study (Kothari, 2004). A total of 17 key informants were interviewed including, one village leader in each village, two environmental officers from National Environmental Management Council (NEMC) Kigoma office, one fisheries officer in each village and one citizen scientists group leader in each village. These key informants were selected based on their knowledge and concerns about water quality in the Lake Tanganyika and willingness to provide necessary in-depth information to answer research questions. All FGDs and KIIs sessions were audio recorded to capture information during discussion.

### Data analysis and Presentation

Qualitative information from the FGDs and KIIs were analyzed through content analysis (Erlingsson and Brysiewicz, 2017). Content analysis is a research technique for narrative and non-numeric data collected in form of speeches or quotes. In this case the researcher is required to identify and interpret the related messages within a given set of information and produce meaningful insights from the phenomenon under study (Bengtsson, 2016; Erlingsson and Brysiewicz, 2017).

Quantitative data gathered from individual questionnaire survey were sorted using Microsoft Excel, and then coded in SPSS statistical software, version 27 for descriptive and inferential statistical analysis. Descriptive statistics was applied to determine of percentage response of both citizen scientists (*n* = 110) and non-citizen scientists (*n* = 110) on their socioeconomic and demographic characteristics, personal and social expectation for engagement in citizen science activities as well as their preferred incentives. A chi-squared test of independence was used to compare the motivational factors for participation in citizen science water quality monitoring of the two groups of citizen scientists and non-citizen scientists. One-way ANOVA was applied to compare these attributes mean score between citizen scientists and non-citizen scientists’ groups.

Logistic regression analysis was employed to determine the influence of socio-economic and demographic characteristics on respondents’ willingness to engage in current/future citizen science activities in the study area. In this analysis, the two groups of citizen scientists (110) and non-citizen scientists (*n* = 110) were pooled together to increase the strength of the association between the predictor and response variables that fit the logit model (Field, 2009). The logistic model was used to predict the probability of an individual’s willingness to participate in citizen water quality monitoring activities based on their sociodemographic profiles (Comoé and Siegrist, 2015). The variables included in the model were defined on the basis of the link between a binary dependent variable and independent (predictor) variables, which were either categorical or continuous (Karasmanaki et al., 2019; Kitula et al., 2015; Kostakis and Sardianou, 2012; Midi et al., 2010) (Table 1). Independent variables entered in the logistic equation were: respondent’s age in years, gender (male or female), household size, education level (informal, primary, secondary and college), residence time (years) individual have lived in the area, marital status (single, married, widow and divorced), head of household (yes or no) and monthly income (estimated monthly income earned per individual). Dummy variables for independent data such as marital status and education level were selected as a reference group (Table 1). The dependent variable was willingness to participate in the future citizen science activities in the area (yes or no).

**Table 1:** Dependent and independent variables used in the logistic regression model analysis

Variables		Descriptions	Coded value
Dependent	Willingness to participate in future citizen science activities	If individual is willing to participate in future citizen science activities in the area	1 = Yes
			0 = No
Independent variables	Age	Respondents age in years	Continuous
	Gender	Respondent’s gender	1 = Male
			0 = Female
	Household size	Number of people in respondent’s household	Continuous
	Education level	Highest level attained by individual based on: educated (primary, secondary, college)	1 = educated
			0 = non-educated
		Non-educated (informal)	
	Residence time in the village	Length of time in years, the respondent has lived in the area	Continuous
	Head of household	If the respondent is head of household	1=Yes
			0 = No
	Marital status	If the individual respondent is married (married) or not married (single, divorce, widow)	1 = Married
			0 = Not Married
	Monthly income	Monthly income earned by individual respondent	Continuous

## Results

### Socioeconomic and Demographic Characteristics of Respondents

The majority of respondents were male (69% and 74%) for citizen scientists and non-citizen scientists respectively (Table 2). The dominating age group (41% and 36% for citizen and non-citizen scientists respectively) was between 29 to 39 years old (Table 2). For education level, 64% of citizen scientists respondents and 71% of non-citizen scientists had attained primary education (Table 2). This study revealed that most of respondents were household heads (73% and 83% for citizen scientists and non-citizen scientists respectively). Fishing was the dominant occupation accounting the overall response of 49% (48% citizen scientists and 50% non-citizen scientists (Table 2). Most of citizen scientists and non-citizen scientists have lived in the area for more than 20 years (53%). In terms of marital status, 51% of citizen scientists and 55% of non-citizen scientists were married (Table 2). The monthly income earned by most of the respondents in the survey was less than 200,000 Tanzania Shillings ($90 USD), for 67% and 73% for citizen scientists and non-citizen scientists respectively (Table 2).

**Table 2:** Socioeconomic and demographic characteristics of respondents in the study area

		Citizen scientists (CS, *n* = 110)		Non-Citizen scientists (Non-CS, *n* = 110)	overall
Variable	Group	Response in %			
Gender	Male	69	74		71
	Female	31	26		29
Age	18–28	22	18		20
	29–39	41	36		39
	40–50	21	25		23
	51–61	10	14		12
	>61	6	7		6
Education level	Informal	0	3		1
	primary	64	71		67
	secondary	25	13		19
	College	11	14		12
Household size	1–5	27	20		24
	6–10	56	63		60
	>10	16	17		17
Head of household	Yes	73	83		78
	No	27	17		22
Main occupation	Fishing	48	50		49
	Farming	20	18		19
	Small business	13	9		11
	Public servant	1	5		4
	Fish processing	14	10		12
	Fish vending	4	7		5
Residence time	<10 years	16	19		18
	10–20 years	31	27		29
	>20 years	53	54		53
Marital status	Single	28	30		29
	Married	51	55		53
	Divorced	15	10		13
	Widow	5	5		5
Monthly income	<200,000	67	73		70
	200,000–300,000	25	17		21
	>300,000	8	10		9

### Influence of Socio-demographic Characteristics

Logistic regression indicated individuals’ willingness to participate in future citizen science activities were significantly influenced by their socio-demographic characteristics (*p* = 0.044). Higher model correctness with prediction success of 84% was observed. The model variation showed that the dependent variable can be explained by independent variables by 63% (Nagelkerke R^2^ = 0.63, Cox and Snell R^2^ = 0.47 and −2Log likelihood = 161.30). Willingness to participate was favoured by increased age, being male, low monthly income, longer permanence in the village and being the head of household (Table 3). An increase in individual monthly income reduced the likelihood of individuals participating in future citizen science activities (Table 3).

**Table 3:** Results of the logistic regression model showing the relative influence of socio-demographic characteristics of respondents on willingness to participate in water quality monitoring along Lake Tanganyika

Variables	β	S.E.	Sig.	Exp (β))
Age of Respondent	1.229	0.424	0.004*	3.419
Gender of Respondent	1.743	0.420	<0.001*	5.717
Household size	0.457	0.404	0.258	1.580
Education level	0.361	0.423	0.394	1.435
Monthly income	−1.234	0.420	0.003*	0.291
Residence time in the village	1.855	0.413	<0.001*	6.393
Head of household	1.314	0.419	0.002*	3.720
Marital status	0.458	0.418	0.273	1.582
Constant	−3.318	0.688	0.001	0.036

Asterisk indicates significant factors at *p* < 0.05, Standard Error (SE), regression coefficient (β), a negative sign (−) of β indicates a decrease in the odds of respondent’s willingness to engage in water quality monitoring (Exp(β) with a unit increase in the variable

### Motivation Factors

**Fig. 2:**
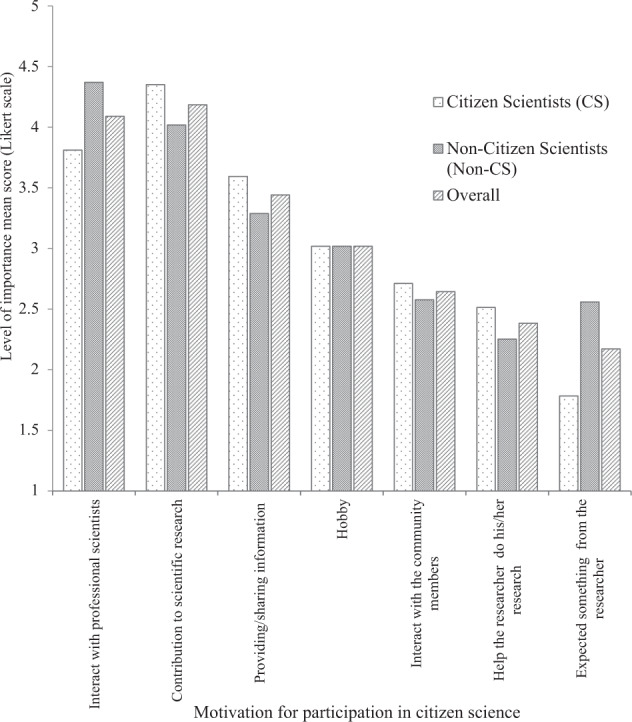
Mean score on the level of importance in motivation factors for individual participation in citizen science water quality monitoring according to two groups of citizen scientists (CS) (*n* = 110) and non-CS (non-citizen scientists) (*n* = 110) in the study area. Likert scale are defined by; 1 = very low importance, 2 = low importance, 3 = medium importance, 4 = high importance and 5 = very high importance

The mean score of all motivation factors for participation in citizen science water quality monitoring differed significantly between citizen scientists and non-citizen scientists (χ^2^ = 17.02, *p* < 0.001). Interaction with professional scientists and contribution to scientific research were cited as the most important motivation factors (4.4 mean score) for participation in water quality monitoring by both non-citizen scientists and citizen scientists (Fig. 2). This motivation was also supported by narratives from both FGDs and KIIs, as expressed in the following statements:

*“I would like to participate and being involved in citizen science water quality monitoring because is the only chance I have to connect with professionals and learn about aquatic environments, the closer you are to the bees the more the chance of getting honey”. I believe that when you are close to professionals like environment conservationists, aquatic researchers the more you gain knowledge”*. (FGDs/Non-citizen scientists/Kibirizi. Likewise, one of the citizen scientist group leaders (Karago) explained that *“I’m motivated to participate in citizen science activities because I feel more honored when given the opportunity to explain my feelings about my surrounding environment and contribute my time, labour and knowledge to scientific research activities in my area”*.

### Expectation for Participation in Citizen Science Monitoring

**Fig. 3:**
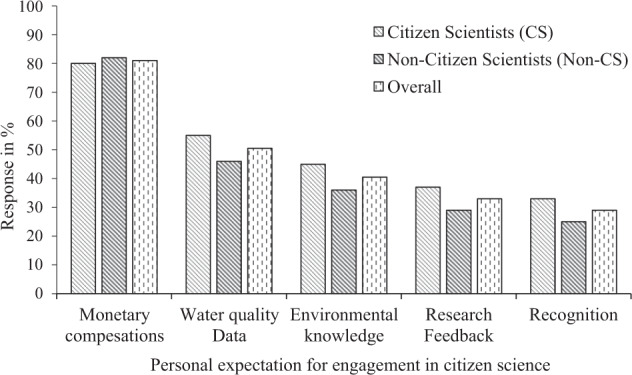
Percentage response on personal expectation for individual engagement and participation in citizen science water quality monitoring in the study area based on two groups of citizen scientists (CS) (*n* = 110) and non-citizen scientists (*n* = 110)

**Fig. 4:**
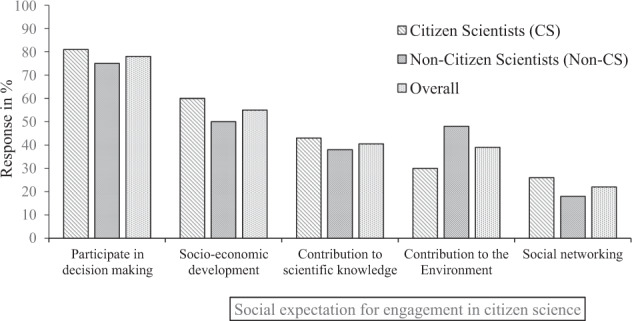
Percentage response on social expectation for individual engagement and participation in citizen science water quality monitoring in the study area based on two groups of citizen scientists (CS) (*n* = 110) and non-citizen scientists (*n* = 110)

Findings from individual surveys revealed that most of citizen scientists and non-citizen scientists interviewed were expecting some monetary compensation for participating in water quality monitoring in the study area (80% and 82% for citizen and non-citizen scientists) (Fig. 3). In addition, most citizen scientist respondents (81%) and non-citizen scientists (78%) were expecting to participate in decision making regarding their local environment (Fig. 4). These results were also corroborated from FGDs and KIIs whereby both groups:

*“When I participate in citizen science water quality monitoring activities, I expect to be paid for my time and knowledge I contribute to water quality monitoring whether it is monthly or daily as I will not do other income generating activities during that time”* (FGDs/non-citizen scientists/Mwamgongo). *One of the village leaders added that: “We are a complete society, if we participate in citizen science activities, we expect our people in this village to participate in decision making concerning our waters and no one else to decide for us”* (KIIs/Ujiji).

### Incentives for Engagement in Citizen Science Water Quality Monitoring Activities

**Fig. 5:**
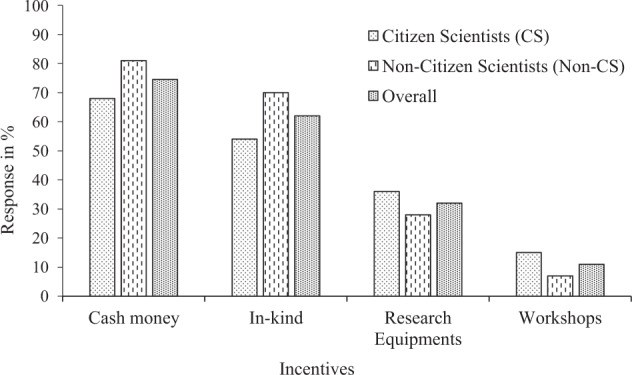
Types of incentives preferred by citizen scientists (*n* = 110) and non-citizen scientists (*n* = 110) for participating in water quality monitoring activities in the study area

Results from individual surveys revealed that cash incentives were the most preferred incentive for participation (68% and 74.5% for citizen scientists and non-citizen scientists respectively) (Fig. 5). On the basis of the results from FGDs and KIIs, it was also confirmed that cash money was the highest ranked incentive for participation in citizen science water quality monitoring activities for both citizen scientists and non-citizen scientists (Table 4).

**Table 4:** Qualitative summary from FGDs and KIIs of the most preferred incentives for participation in citizen science water quality monitoring activities in the study area

Rank	Citizen scientists and non-citizen scientists’ groups preferred incentives
(Arranged from the most important to the least important)	Cash money
	Caps, T-shirts, pens, pencils, bags, thank people verbally, prizes, construction of community based environmental monitoring offices, schools and road constructions.
	Thermometers, water test-kits, turbidity tubes, waterproof research proofs, raincoats, gloves, smartphones, notebooks, cameras and watches.
	Workshops, scientific meeting invitations.

### Sustainability for Citizen Science Water Quality Monitoring Activities in the Study Area

Our study reported that, community members in Lake Tanganyika were willing to continue monitoring water quality in Lake Tanganyika over the long-term. Sharing water quality results with other community members was cited as the important sustainable way with higher mean score (4.2 ± 0.73 and 4.0 ± 0.83 for citizen scientists and non-citizen scientists respectively) (Table 5). These results were also evident in the FGDs and KIIs:

*“Sharing is caring, to my side I would like to share water quality data obtained from my research to other fellows in the community. By this way I believe that, other people and their families will be interested and attracted and join in future aquatic research activities as I do”*, (FGDs/citizen scientist/Ilagala). *Similarly, one of the environmental officer indicated that, “if you want to make this citizen science monitoring approach sustainable in this area, people who are already citizen scientists must first share what they have achieved in their monitoring with other communities to raise awareness of what is happening in the environment and the word will spread all over the society, and more people will be ready to participate in the upcoming citizen science activities”* (KIIs/non-citizen scientist/Kibirizi).

**Table 5:** Approaches suggested to ensure sustainability of citizen science water quality monitoring based on individual surveys in the study area (mean score ± standard deviation)

Statement	Citizen Scientists (CS)	Non-Citizen Scientists (Non-CS)	Overall
Sharing water quality results with other community members	4.2 ± 0.73	4.0 ± 0.83	4.1 ± 0.14
Sharing the experience of doing science to other who don’t participate yet	3.68 ± 0.58	3.58 ± 0.54	3.63 ± 0.07
Providing/sharing information	**3.38** ± 0.76	**3.63** ± 0.79	3.505 ± 0.17
Collaboration with other citizen scientists	**3.0** ± 0.95	**2.44** ± 1.0	2.72 ± 0.39
Continue monitoring and inform decision makers	**2.75** ± 0.67	**2.27** ± 2.77	2.51 ± 0.33
Accompany the researcher on future scientific activities	**2.3** ± 0.57	**2.87** ± 1.1	2.58 ± 0.4

Bold values within rows show significantly different responses between participating citizen scientists and non participants (*p* < 0.05). Likert score scale: 1 = very low importance, 2 = low importance, 3 medium importance, 4 = high importance and 5 = very high importance

## Discussion

### Influence of Socio-demographic Characteristics

Socio-demographic characteristics were the most important predictor of willingness to participate in citizen science activities. The older the person, the more likely is he/she is likely to engage in citizen science activities. In our study, this was confirmed as most respondents between 29–39 years old (70%) were willing to be involved in future citizen science activities. People of this age have families as well as the time and energy to contribute to managing their environment. Our study supports previous studies done by Musyoki et al. (2016), where family aged participants (36–50 years of age) were the most (41%) willing to participate in Community Forest Associations to improve forest cover and livelihood in Kenya as well as other studies showing this age groups as more concerned with the environment (Howard and Parsons, 2006; Wiener et al., 2016). We note that the snowball sampling method used to recruit non-citizen scientists presents a bias reflecting the social network of the original citizen scientists that were originally randomly selected (Marcus et al., 2017).

Men had a higher willingness to participate in citizen science activities. Men are traditional breadwinners in most families in Tanzania, and tend to participate more in environmental stewardship to secure livelihood of their families (Nyangoko et al. 2022). In the present study, we observed that men were more often the family and society decision makers compared to women, as also seen by Tindall et al. (2003). Women are more likely to express concern for the environment but less likely to be involved in environmental activism and less likely to express their willingness to participate. This is also supported by the result that being the head of household had a positive influence on individual willingness to participate in citizen science. Being the head of household increases the need to find options to increase the family quality of life, of which the environment is important. Komba and Muchapondwa (2017) reported that being household head also increased willingness to participate in Reducing Emissions from Deforestation and forest Degradation (REDD^+^) programmes in Tanzania.

Interestingly, people with a low monthly income were more likely to be willing to participate in citizen science activities. This influence maybe linked with either the intention to increase their income through participation in citizen science or because they desire to protect their environment and increase their quality of life but have limited options, as suggested by Shao et al. (2018). People with higher incomes might recognise that they have more capacity to protect themselves and their families from environmental threats (Zheng and Kahn, 2008).

Education level was not a significant factor for participation. It should be noted that this may the result of the limited variance of the participating population, typical of the population of these villages, dominated by a single education level (primary) (67%). Many studies have shown that literate people are more aware of potential benefits obtained from well-managed water resources (Musyoki et al., 2016). Education improves the development of general knowledge, and consequently, the understanding of one’s responsibility towards the environment.

We also observed that key informants play an important role in mobilizing participation in citizen science (Van De Gevel et al., 2020), In several villages, fisheries officers were instrumental in mobilizing people to participate in the project activities.

### Citizen Scientists’ Motivation

Improving individual reputation and learning new skills were the most prominent motives for citizen science participation (West and Pateman, 2016). These more egoistic motivations were different than previous studies (Geoghegan et al., 2016; Maund et al., 2020; McDougle et al., 2011) associating participation to more altruistic interests. For example, the study by Moczek et al. (2021) showed volunteers were largely driven by altruistic motivations like social responsibility and nature conservation values, while egoistic functions like enhancement, work-life balance, and career motivation received the lowest levels of agreement.

Our study showed a desire to be included and contribute to scientific research can drive people towards environmental activism. These results were similar to those reported by other studies (Curtis, 2015; Domroese and Johnson, 2017; Raddick et al., 2013b). This suggests that program design should make clear the significance of participants’ contributions, demonstrating the extent to which their data contribute to positive environmental actions (Land-Zandstra et al., 2016).

Our study revealed that most of the respondents (citizen scientists and non-citizen scientists) were looking for income generation from participation. This is a clear difference with most stuedies exploring participation in citizen science in developed countries. However, similar findings were also reported by Paul et al. (2020) in Nepal, who showed that nearly all participants referred to financial compensation as the greatest source of motivation for contributing to rainfall monitoring, and in the study by in Larson et al., (2016) in Sierra Leone where nearly half of citizen scientists mentioned financial compensation as a major motivation for environmental activities. The difference in living standard and economic status between respondents in different studies clearly influences this divergence. In developed countries, people can consider citizen science as an opportunity to spend time in nature with their families and friends and increase their relationship with natural environment (Rotman et al., 2014). Our study suggests that for people in low-income countries, this opportunity is less important.

Citizen scientists involved in Lake Tanganyika water quality program were given a limited monetary incentive to buy airtime vouchers, necessary for data transmission. Although monetary incentives can increase extrinsic motivation, if the amount is too high, citizen scientists may regard this incentive as a control factor with impacts on self-esteem and self-determination (Capdevila et al., 2020). Our results are in-line with those of Restuccia et al. (2016) who suggested that, to encourage as many actors as possible to engage in environmental activities, multiple compensation models should be considered, not only monetary, such as attractive resources such as T-shirts, caps, prizes, certificates and shop discount cards.

### Sustainable Citizen Science Water Quality Monitoring in Lake Tanganyika

Considering the limitations to water quality monitoring in developing countries like Tanzania, a citizen science approach presents benefits to agencies as well as the participating communities. Our study identified that a key motivation for participation was knowledge sharing of the results with others, suggested by both citizen scientists and non-citizen scientist respondents. This implies that through experience sharing, citizen scientists pass knowledge among their friends, family, and colleagues by discussing the issues they care about through a wide range of social linkages, generating an extensive impact (Dean et al., 2018; Johnson et al., 2014). When citizens produce and gain scientific knowledge about the environment, they have the opportunity to transform their own relationship with nature and ensure sustainability of monitoring initiatives (Johnson et al., 2014). This was particularly relevant in the present study, as participants cited the importance of their involvement in environmental decision-making processes as a result of their activities as citizen scientists, also noted by Pocock et al., (2019).

## Conclusions

The socio-demographic characteristics were found to be key factors in individual willingness to participate in citizen science activities. For proper recruitment and retention of citizen scientists in ongoing and future monitoring activities, participants with families, head of households and individuals with low monthly income present the most willing participants.

To support motivation, citizen science projects should connect community members to scientists and specify their contribution to new scientific knowledge. The significance of citizen scientists contributions should be demonstrated, showing the extent to which their data and observations contribute to lake management and meeting water quality monitoring goals (Lee et al., 2018).

While any one citizen science project may not accomplish all the participants’ expectations, integrating the expectations of participants within the project design is recommended. Identification and provision of incentives will facilitate participation and favor their long-term participation (Bonaccorsi and Rossi, 2003).

Citizen science programs in developing countries should take into account monetary incentives, but not only, as many of the same incentives shown to be important in citizen science in developed countries were also identified in the present study.

## Data Availability

All citizen science data are open data, available on the FWW website https://www.freshwaterwatch.org/. All survey and questionnaire data are available from the corresponding authors on reasonable request.

## Appendix 1: Motivational Factors for participation in citizen science Water Quality Monitoring (Individual Questionnare survey)

Theme 1: Socioeconomic characteristics of respondents (please, circle the relevant answer)

1. Gender

1 = Male 2 = Female

2. How old are you?

1 = 18–28 2 = 29–39 3 = 39–49

4 = 49–59 5 = 60–69 6 = >69

3. What is your highest level of education?………………

4. Head of household?

1 = Yes 2 = No

5. Main economic activity of the household head

1 = 1 = fishing 2 = Agriculture3 = small business

4 = fisheries officer 5 = village leader 6 = fish processor

7. = Environment officer 8 = others, specify

6. Number of household members…………..….

7. Your daily use of Lake Tanganyika……………….., …………….., ……………………..

8. What is your main economic activity (if different from head of household)?

1 = fishing 2 = Agriculture 3 = small business

4 = fisheries officer 5 = village leader 6 = fish processor

7. Environment officer 8 = others, specify

9. How much is your monthly income? (T.shs)

1 = less than 200000 2 = 200,000–300,000 3 = More than 300,000.

10. Do you know how to use smartphone?

1 = yes 2 = no

11. Do you own a smartphone?

1 = Yes 2 = No

12. Do you have frequent access to the lake shore?

1 = yes 2 = No

13. Are you a member of any organization?

1 = yes 2 = No

14. If yes please mention the organization you are involved………………………….

**Theme 2:** Motivation factors for participation in citizen science water quality monitoring.

**Table:**

15a).	Are you willing to participate in citizen science based water quality monitoring in your area?	[] Yes [] No
15b).	If No, why?……………………………………………………………………………………………………	

If you responded to “Yes” in the above Question

Please rate each of the following motivations according to how important they are to your willingness to support and continue your participation in citizen science water quality monitoring on Lake Tanganyika.

**1** = very low importance ***2*** = low importance ***3*** = medium importance ***4*** = High importance ***5*** = very high importance

**Table:**

	***Statement***	***Rank of how important***					
16.	To contribute to scientific research activities	*1*	*2*	*3*		*4*	*5*
17.	It’s my hobby to do such kind of activities	*1*	*2*	*3*		*4*	*5*
18.	Providing/sharing information is interesting to me	*1*	*2*	*3*		*4*	*5*
19.a).	I expect something in return from the researcher:	*1*	*2*	*3*		*4*	*5*
19.b)	What do you expect in return for conducting such activity?…………………………						
20.	To interact/network with researchers and professional scientists	*1*	*2*	*3*	*4*		*5*
21.	To interact/network with the community members	*1*	*2*	*3*	*4*		*5*
22.	To help the researcher to get his/her research done	*1*	*2*	*3*	*4*		*5*
23.	If you have other motivation factor apart which is not mentioned above, please specify	………………………………………………………………………………………………..					

24a. If you are asked to share information about water quality in your area in the future, would you expect recompense? Yes [] No []

24b. If yes, what kind of recompense? Specify …………………….………………………………

25a. Would you like to make water quality monitoring citizen science sustainable in Lake Tanganyika and other surrounding water bodies. Yes [] No []

25b. In case of *“*yes*”*, how could you make it sustainable? Rank from the most important statement to the least important

(**1** = ***Not important at all, 2*** = ***Not important 3*** = ***Neutral 4*** = ***Important 5*** = ***Very important)***

[] Give more information and instruction about my participation in water quality monitoring.

[] Sharing my environmental results with others

[] Collaborate with others who participate in Lake Tanganyika citizen science

[] Explain the experience of doing science to others who don’t participate yet

[] Accompany the researchers on other future scientific activities (e.g. to go to a new community and explain the results)

[] Somethings else: …………………………………………………………………………

## Appendix 2: Motivational factors for participation in citizen science water quality monitoring (Focus Group discussion checklist Questions)

Part 1: Citizen scientists

1. How far do you understand about water quality conditions in Lake Tanganyika?
2. How far are the community members in this area are willing to monitor water quality?
3. What are the motivational factors for participating in citizen science in your area?
4. What is the people expectation for involving in citizen science what quality monitoring in Lake Tanganyika?
5. What kind of incentives would you prefer most for involvement in water quality monitoring?
6. How would citizen science groups in Lake Tanganyika villages ensure the sustainability of citizen science approach in monitoring water quality?

Part 2: Non-citizen scientists

1. How do you know about citizen science water quality monitoring in your area?
2. Would other people in this area who are not part of water quality monitoring program willing to participate in the future citizen science programs?
3. What do you think are the most motivation factors for them to participate in Lake Tanganyika water quality monitoring?
4. What type of incentive will be appropriate for them for participating in the program?
5. What do you think are the most ways of making this citizen science approach more sustainable in Lake Tanganyika and other waterbodies?

## References

[CR1] Alender B (2016). Understanding volunteer motivations to participate in citizen science projects: a deeper look at water quality monitoring. J Sci Commun.

[CR2] Asah ST, Lenentine MM, Blahna DJ (2014). Benefits of urban landscape eco-volunteerism: Mixed methods segmentation analysis and implications for volunteer retention. Landsc Urban Plan.

[CR92] Asingizwe D, Poortvliet PM, Koenraadt CJ, van Vliet AJ, Ingabire CM, Mutesa L, Leeuwis C (2020). Why (not) participate in citizen science? Motivational factors and barriers to participate in a citizen science program for malaria control in Rwanda.. PLoS One.

[CR85] Bela G, Peltola T, Young JC, Balázs B, Arpin I, Pataki G, Hauck J, Kelemen E, Kopperoinen L, Van Herzele A (2016). Learning and the transformative potential of citizen science.. Conserv Biol.

[CR5] Bengtsson M (2016). How to plan and perform a qualitative study using content analysis. NursingPlus open.

[CR6] Beza E, Steinke J, Van Etten J, Reidsma P, Fadda C, Mittra S, Mathur P, Kooistra L (2017). What are the prospects for citizen science in agriculture? Evidence from three continents on motivation and mobile telephone use of resource-poor farmers. PLoS One.

[CR7] Bishop IJ, Warner S, van Noordwijk TC, Nyoni FC, Loiselle SJS (2020). Citizen Science Monitoring for Sustainable Development Goal Indicator 6.3. 2 in England and Zambia. Sustainability.

[CR8] Bonaccorsi A, Rossi C (2003). Why open source software can succeed. Res policy.

[CR9] Bonney R, Cooper CB, Dickinson J, Kelling S, Phillips T, Rosenberg KV, Shirk J (2009). Citizen science: a developing tool for expanding science knowledge and scientific literacy. BioScience.

[CR10] Budhathoki NR, Haythornthwaite C (2013). Motivation for open collaboration: Crowd and community models and the case of OpenStreetMap. Am Behav Scientist.

[CR11] Capdevila ASL, Kokimova A, Ray SS, Avellán T, Kim J, Kirschke S (2020). Success factors for citizen science projects in water quality monitoring. Sci Total Environ.

[CR12] Cohen N, Arieli T (2011). Field research in conflict environments: Methodological challenges and snowball sampling. J peace Res.

[CR13] Comoé H, Siegrist M (2015). Relevant drivers of farmers’ decision behavior regarding their adaptation to climate change: a case study of two regions in Côte d’Ivoire. Mitig Adapt Strateg Glob Change.

[CR14] Curtis V (2015). Motivation to participate in an online citizen science game: A study of Foldit. Sci Commun.

[CR15] Dean AJ, Church EK, Loder J, Fielding KS, Wilson KA (2018). How do marine and coastal citizen science experiences foster environmental engagement?. J Environ Manag.

[CR16] Domroese MC, Johnson EA (2017). Why watch bees? Motivations of citizen science volunteers in the Great Pollinator Project. Biol Conserv.

[CR17] Erlingsson C, Brysiewicz P (2017). A hands-on guide to doing content analysis. Afr J Emerg Med.

[CR18] Field A (2009). Discopering Statistics Using SPSS, Thrid Edition. Fritz, S., See, L., Carlson, T., Haklay, M., Oliver, J.L., Fraisl, D., Mondardini, R., Brocklehurst, M., Shanley, L.A., Schade, S. and Wehn, U., 2019. Citizen science and the United Nations sustainable development goals. Nature. Sustainability.

[CR19] Fraisl D, Campbell J, See L, Wehn U, Wardlaw J, Gold M, Moorthy I, Arias R, Piera J, Oliver JL, Masó J (2020). Mapping citizen science contributions to the UN sustainable development goals. Sustainability. Science.

[CR20] Ganzevoort W, van den Born RJ, Halffman W, Turnhout S (2017). Sharing biodiversity data: citizen scientists’ concerns and motivations. Biodivers Conserv.

[CR21] Geoghegan H, Dyke A, Pateman R, West S, Everett G (2016) Understanding motivations for citizen science. Final report on behalf of UKEOF, University of Reading, Stockholm Environment Institute (University of York) and University of the West of England

[CR22] Hadj-Hammou J, Loiselle S, Ophof D, Thornhill I (2017). Getting the full picture: Assessing the complementarity of citizen science and agency monitoring data. PLoS One.

[CR23] Howard C, Parsons E (2006). Attitudes of Scottish city inhabitants to cetacean conservation. Biodivers Conserv J.

[CR24] Hughes RN, Hughes DJ, Smith IP (2014). Citizen scientists and marine research: volunteer participants, their contributions, and projection for the future. Oceanogr Mar Biol: Annu Rev.

[CR25] Hyder K, Wright S, Kirby M, Brant J (2017). The role of citizen science in monitoring small-scale pollution events. Mar Pollut Bull.

[CR26] Johnson MF, Hannah C, Acton L, Popovici R, Karanth KK, Weinthal E (2014). Network environmentalism: Citizen scientists as agents for environmental advocacy. Glob Environ Change.

[CR27] Karasmanaki E, Galatsidas S, Tsantopoulos G (2019). An investigation of factors affecting the willingness to invest in renewables among environmental students: A logistic regression approach. Sustainability.

[CR28] Kitula R, Larwanou M, Munishi P, Muoghalu J, Popoola L (2015). Climate vulnerability of biophysical systems in different forest types and coastal wetlands in Africa: a synthesis. Int Forestry Rev.

[CR86] Komba C, Muchapondwa E (2017). An analysis of factors affecting household willingness to participate in the REDD+ programme in Tanzania.. Clim Dev.

[CR31] Kostakis I, Sardianou E (2012). Which factors affect the willingness of tourists to pay for renewable energy?. Renew Energy.

[CR32] Kothari CR (2004) Research methodology: Methods and techniques. New Age International

[CR33] Kotrlik J, Higgins C (2001). Organizational research: Determining appropriate sample size in survey research appropriate sample size in survey research. Inf Technol, Learn, Perform J.

[CR34] Kragh G (2016). The motivations of volunteers in citizen science. Environ Scientist.

[CR35] Land-Zandstra AM, van Beusekom MM, Koppeschaar CE, van den Broek JM (2016). Motivation and learning impact of Dutch flu-trackers. J Sci Commun.

[CR36] Larson LR, Conway AL, Hernandez SM, Carroll JP (2016). Human-wildlife conflict, conservation attitudes, and a potential role for citizen science in Sierra Leone, Africa. Conserv Soc.

[CR37] Lee TK, Crowston K, Harandi M, Østerlund C, Miller G (2018). Appealing to different motivations in a message to recruit citizen scientists: results of a field experiment. J Sci Commun.

[CR38] Lewandowski EJ, Oberhauser KS (2017). Butterfly citizen scientists in the United States increase their engagement in conservation. Biol Conserv.

[CR39] Loiselle AS, Gasparini Fernandes Cunha D, Shupe S, Valiente E, Rocha L, Heasley E, Belmont PP, Baruch A (2016). Micro and macroscale drivers of nutrient concentrations in urban streams in South. Cent North Am PLoS One.

[CR40] Luther K, Counts S, Stecher KB, Hoff A, Johns P (2009) Pathfinder: an online collaboration environment for citizen scientists. In Proceedings of the SIGCHI Conference on Human Factors in Computing Systems (pp. 239–248)

[CR41] Marcus B, Weigelt O, Hergert J, Gurt J, Gelléri P (2017). The use of snowball sampling for multi source organizational research: Some cause for concern. Pers Psychol.

[CR42] Marshall MN (1996). Sampling for qualitative research. Fam Pract.

[CR43] Maund PR, Irvine KN, Lawson B, Steadman J, Risely K, Cunningham AA, Davies ZG (2020). What motivates the masses: Understanding why people contribute to conservation citizen science projects. Biol Conserv.

[CR44] Mayoma BS, Mjumira IS, Efudala A, Syberg K, Khan FR (2019). Collection of anthropogenic litter from the shores of Lake Malawi: characterization of plastic debris and the implications of public involvement in the African Great Lakes. Toxics.

[CR45] McDougle LM, Greenspan I, Handy F (2011). Generation green: understanding the motivations and mechanisms influencing young adults’ environmental volunteering. Int J Nonprofit Voluntary Sect Mark.

[CR46] Measham TG, Barnett GB (2008). Environmental volunteering: Motivations, modes and outcomes. Aust Geographer.

[CR47] Midi H, Sarkar SK, Rana S (2010). Collinearity diagnostics of binary logistic regression model. J Interdiscip Math.

[CR87] Moczek N, Nuss M, Köhler JK (2021). Volunteering in the citizen science project “insects of saxony”—the larger the island of knowledge, thelonger the bank of questions.. Insects.

[CR49] Moshi HA, Shilla DA, Kimirei IA, O’Reilly C, Clymans W, Bishop I, Loiselle SA (2022). Community monitoring of coliform pollution in Lake Tanganyika. PLoS One.

[CR50] Musyoki JK, Mugwe J, Mutundu K, Muchiri M (2016). Factors influencing level of participation of community forest associations in management forests in Kenya. J Sustain Forestry.

[CR51] NBS (2013) Tanzania National Bureau of statistics; 2012 population and housing census

[CR52] Nijhawan A, Howard G (2022). Associations between climate variables and water quality in low-and middle-income countries: A scoping review. Water Res.

[CR53] Njue N, Kroese JS, Gräf J, Jacobs S, Weeser B, Breuer L, Rufino M (2019). Citizen science in hydrological monitoring and ecosystem services management: State of the art and future prospects. Sci Total Environ.

[CR93] Nyangoko BP, Berg H, Mangora MM, Shalli MS, Gullström M (2022). Local perceptions of changes in mangrove ecosystem services and their implications for livelihoods and management in the Rufiji Delta, Tanzania.. Ocean Coast Manag.

[CR55] Paul JD, Cieslik K, Sah N, Shakya P, Parajuli BP, Paudel S, Dewulf A, Buytaert W (2020). Applying citizen science for sustainable development: Rainfall monitoring in western Nepal. Front Water.

[CR57] Pocock MJ, Roy HE, August T, Kuria A, Barasa F, Bett J, Githiru M, Kairo J, Kimani J, Kinuthia W (2019). Developing the global potential of citizen science: Assessing opportunities that benefit people, society and the environment in East Africa. J Appl Ecol.

[CR58] Raddick MJ, Bracey G, Gay PL, Lintott CJ, Cardamone C, Murray P, Schawinski K, Szalay AS, Vandenberg J (2013a). Galaxy Zoo: Motivations of citizen scientists. arXiv preprint arXiv:1303.6886

[CR59] Raddick J, Lintott C, Bamford S, Land K, Locksmith D, Murray P, ... Andreescu D (2008) Galaxy zoo: Motivations of citizen scientists. In American Astronomical Society Meeting Abstracts# 212 (Vol. 212, pp. 40–01)

[CR60] Restuccia F, Das SK, Payton J (2016). Incentive mechanisms for participatory sensing: Survey and research challenges. ACM Trans Sens Netw.

[CR61] Reynolds J, Moelsae H (2000). Lake Tanganyika Regional Fisheries Programme(TREFIP). Environmental impact assessment report. FISHCODE Management. FAO, Rome(Italy).

[CR62] Rotman D, Hammock J, Preece JJ, Boston CL, Hansen DL, Bowser A, He Y (2014) Does motivation in citizen science change with time and culture? In Proceedings of the companion publication of the 17th ACM conference on Computer supported cooperative work & social computing (pp. 229–232)

[CR63] Roy HE, Pocock MJ, Preston CD, Roy DB, Savage J, Tweddle JC, Robinson LD (2012) Understanding citizen science and environmental monitoring: final report on behalf of UK Environmental Observation Framework

[CR64] Rufino MC, Weeser B, Stenfert Kroese J, Njue N, Gräf J, Jacobs S, ... Breuer L (2018) Citizen scientists monitor water quantity and quality in Kenya (Vol. 230). CIFOR

[CR88] Ryan RL, Kaplan R, Grese RE (2001). Predicting volunteer commitment in environmental stewardship programmes.. J Environ Plan Manag.

[CR67] Sauermann H, Vohland K, Antoniou V, Balázs B, Göbel C, Karatzas K, Mooney P, Perelló J, Ponti M, Samson R, Winter S (2020). Citizen science and sustainability transitions. Res Policy.

[CR68] Schade S, Pelacho M, van Noordwijk T, Vohland K, Hecker S, Manzoni M (2021) Citizen science and policy. The science of citizen science pp. 351–371

[CR69] Seeberger A (2014) There’s no such thing as free labor: Evaluating citizen science volunteer motivations (Doctoral dissertation, University of Colorado at Boulder)

[CR89] Shao S, Tian Z, Fan M (2018). Do the rich have stronger willingness to pay for environmental protection? New evidence from a survey in China.. World Dev.

[CR72] Sutherland WJ, Roy DB, Amano T (2015). An agenda for the future of biological recording for ecological monitoring and citizen science. Biol J Linn Soc.

[CR73] Thornhill I, Loiselle S, Clymans W, van Noordwijk C (2019). How citizen scientists can enrich freshwater science as contributors, collaborators, and co-creators. Freshw Sci.

[CR90] Tindall DB, Davies S, Mauboules C (2003). Activism and conservation behavior in an environmental movement: The contradictory effects of gender.. Soc Nat Resour.

[CR91] Trochim W (2006) Research methods knowledge base: Introduction to evaluation. Web Center for Social Research Methods

[CR76] Van De Gevel J, van Etten J, Deterding S (2020). Citizen science breathes new life into participatory agricultural research. A review. Agron Sustain Dev.

[CR77] Walker DW, Smigaj M, Tani M (2021). The benefits and negative impacts of citizen science applications to water as experienced by participants and communities. Wiley Interdiscip Rev: Water.

[CR78] Weeser B, Kroese JS, Jacobs S, Njue N, Kemboi Z, Ran A, Rufino M, Breuer L (2018). Citizen science pioneers in Kenya–A crowdsourced approach for hydrological monitoring. Sci Total Environ.

[CR79] West SE, Pateman RM (2016) Recruiting and retaining participants in citizen science: what can be learned from the volunteering literature? Citizen Science: Theory and Practice

[CR80] West SE, Pateman RM, Dyke A (2021) Variations in the motivations of environmental citizen scientists. Citizen Science: Theory and Practice

[CR81] Wiener CS, Manset G, Lemus JD (2016). Ocean use in Hawaii as a predictor of marine conservation interests, beliefs, and willingness to participate: an exploratory study. J Environ Stud Sci.

[CR82] Woodley XM, Lockard M (2016). Womanism and snowball sampling: Engaging marginalized populations in holistic research. Qualitative Rep..

[CR83] Wright DR, Underhill LG, Keene M, Knight AT (2015). Understanding the motivations and satisfactions of volunteers to improve the effectiveness of citizen science programs. Soc Nat Resour.

[CR84] Zheng S, Kahn ME (2008). Land and residential property markets in a booming economy: New evidence from Beijing. J Urban Econ.

